# Research Priorities in Suicide Prevention: Review of Australian Research from 2010–2017 Highlights Continued Need for Intervention Research

**DOI:** 10.3390/ijerph15040807

**Published:** 2018-04-20

**Authors:** Lennart Reifels, Maria Ftanou, Karolina Krysinska, Anna Machlin, Jo Robinson, Jane Pirkis

**Affiliations:** 1 Centre for Mental Health, Melbourne School of Population and Global Health, The University of Melbourne, Carlton 3010, Australia; mftanou@unimelb.edu.au (M.F.); amachlin@unimelb.edu.au (A.M.); j.pirkis@unimelb.edu.au (J.P.); 2 Centre for Primary Health Care and Equity, University of New South Wales, Sydney 2052, Australia; k.krysinska@unsw.edu.au; 3 Orygen, The National Centre of Excellence in Youth Mental Health, Parkville 3052, Australia; jo.robinson@orygen.org.au

**Keywords:** suicide, research, intervention studies

## Abstract

Suicide is a major public health concern in Australia and globally, requiring targeted research efforts to build the evidence base for its effective prevention. We examined current and future priorities in Australian suicide prevention research during the period 2010–2017, and compared these to 1999–2006 baseline data. We classified current research priorities in terms of the type of research published in 424 journal articles and 36 grants and fellowships funded during 2010–2017. A questionnaire administered to 390 stakeholders identified future research priorities. The total number of suicide prevention focussed journal articles and the value of funded grants increased dramatically. Congruent with baseline data, current research priorities in 2010–2017 reflected a strong emphasis on epidemiological studies, while funding for intervention studies declined. This is despite the fact that stakeholders continually identified intervention studies as being the highest future research priority. If we are to make real advances in suicide prevention, we need to know what works, and identify and test effective interventions. This study highlighted the existing dearth and continued need for intervention research. Mechanisms to support future intervention research in suicide prevention are likely to lead to significant gains in knowledge and population health.

## 1. Introduction

Suicide is a major public health problem in Australia and globally. In 2016, there were 2862 suicides in Australia (2149 by males; 713 by females), representing an overall rate of 11.7 per 100,000 (17.8 and 5.8 per 100,000 for males and females, respectively) [1]. In order to address this significant public health concern, it is essential that we gain a better understanding of what does and does not work in suicide prevention. Research and evaluation is therefore crucial for building the evidence base and guiding the choice of effective interventions [2].

In 2006, we compared (then) current research efforts in suicide prevention with stakeholder-identified priorities [3]. We identified suicide-related journal articles published and grants and fellowships funded in Australia in 1999–2006 and classified them according to a pre-determined framework. We asked stakeholders with an interest in suicide prevention where they thought future priorities should lie, using a questionnaire which categorised their responses according to the same framework. The take-home message from this work was that much existing effort had focussed on descriptive epidemiological studies of suicide and suicidal behaviour and that future emphasis should be placed on intervention studies.

The present study repeats the 2006 exercise, examining whether the types of research funded and conducted over the period 2010–2017 have shifted, and whether stakeholder identified future research priorities have changed.

## 2. Materials and Methods

The current study largely replicated the methodology of our original study [3]. We examined current priorities by reviewing journal articles published and grants and fellowships funded during the period 2010–2017. We assessed future priorities by using a questionnaire to elicit stakeholders’ views [4].

We searched three international literature databases (Medline, PsycInfo, and CINAHL) for peer-reviewed articles published during the period from January 2010 to July 2017, using the search terms: suicid* OR self harm OR suicid* attempt* AND Australia. Articles were excluded if they pertained to euthanasia (or assisted suicide); did not include a full abstract; did not involve primary research, a systematic or narrative review, or an evidence-based commentary; and/or did not have a first author with an Australian address, or reported research not conducted in Australia. Each article abstract was examined and classified by a single team member (MF or KK) in consultation with the team leader (JP) wherever necessary (according to an existing research classification framework). To ensure consistency in coding, 14 abstracts were independently coded by two team members (MF and KK) and any discrepancies in the coding of abstracts between the coders were resolved by way of reviewing the code diary and clarifying and refining variable definitions.

We retrieved summary information on grants and fellowships awarded by the National Health and Medical Research Council, Australian Rotary Health, the Australian Research Council, and the Society for Mental Health Research from their respective public website repositories on 11 September 2017. We identified and included all grants and fellowships with an official start (or funding commencement) year in the 2010–2017 period, provided the primary focus of funded activities was on suicide and the research was conducted in Australia.

We administered an online questionnaire between 24 July and 2 October 2017 to seek stakeholders’ views regarding future research priorities. We approached 15 stakeholder groups with a known interest in suicide prevention, including people who conducted, funded, or used suicide prevention research (in clinical practice or in policy-making/planning activities), and people who had been affected by suicide and/or provided advocacy related to suicide prevention. Supplementary Table S1 outlines the targeted stakeholder groups and recruitment strategy. Stakeholders were generally asked to rate the priority they would give to certain types of suicide prevention research in future. This component of the study received ethical approval from the Human Research Ethics Committee at the University of Melbourne (ID: 1749898.1), and yielded responses from 390 respondents. Due to varying recruitment strategies employed, the total denominator was not known and the overall response rate not calculable. Response rates for specific target groups ranged between 5% and 89%. Sixteen per cent of respondents conducted suicide prevention research; 55% used it; 2% were involved in funding it; and 28% had been affected by suicide or were providing advocacy.

We classified suicide prevention research according to a framework based heavily on the one used in our earlier study. The categories in the framework were: research type; suicidal behaviour; suicide method; target group; and setting. This paper focuses on the research type category only. This included the following sub-categories: assessment studies; epidemiological studies; intervention studies; evaluation studies; biological studies; social science studies; and other studies. Findings related to the other categories are reported elsewhere [4].

Data from all three sources were analysed descriptively using SPSS, and charted to identify trends in current and future research priorities from 1999–2006 to 2010–2017. Two-sample tests of proportion were conducted for each data source to examine changes in the proportion of research types across the two time periods.

## 3. Results

During the eight-year period between 2010 and 2017, 424 journal articles were published in the peer-reviewed literature that qualified as research with suicide as the primary focus. During the same period, 36 suicide-related grants and fellowships were funded, totaling $10,580,619. This represents a near doubling of the total number of journal articles published and total amount of research funding awarded when compared to the previous eight-year study period of 1999–2006 (during which 263 articles were published and 36 grants awarded with a total value of $5,839,341) [3].

In 2010–2017, suicide-related journal articles most commonly focussed on descriptive epidemiology (60%), as they had done in 1999–2006 (57%). Journal articles reporting on intervention studies came a distant second (14%), again mirroring the situation in 1999–2006 (18%). Epidemiological studies also dominated the funded grants and fellowships in 2010–2017 (34%), being more common than they had been in 1999–2006 (22%). Significantly less attention was afforded to intervention studies (30%) than had been the case in the previous period (52%).

**Figure 1:**
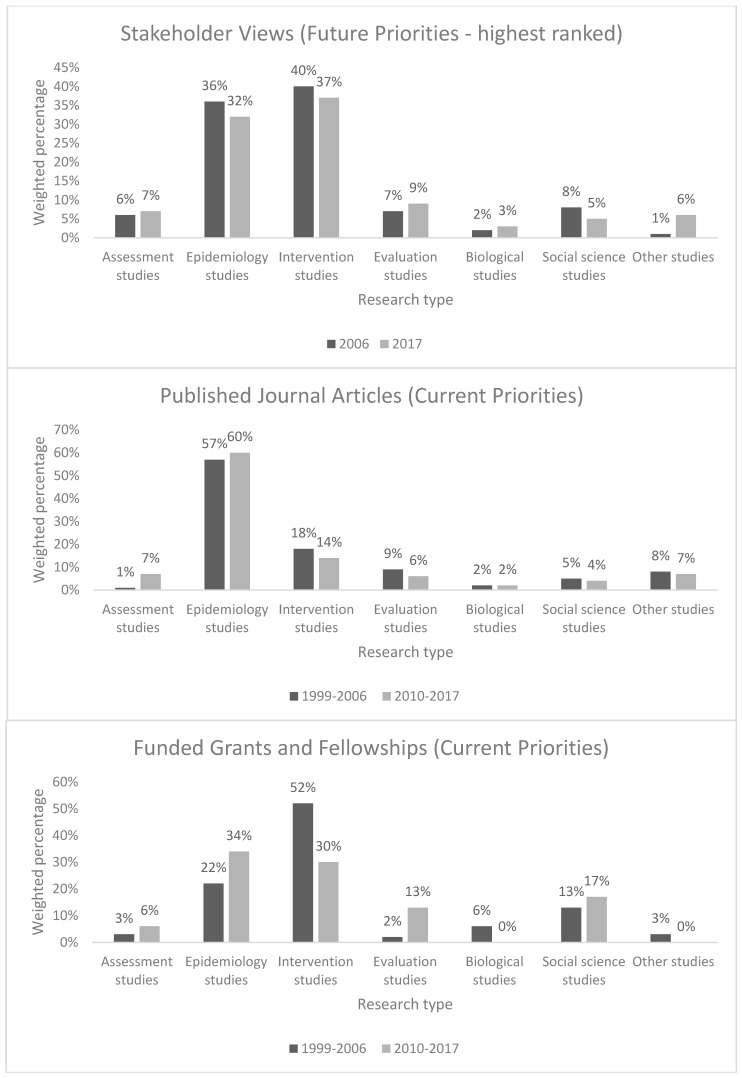
Research priorities identified in published journal articles, funded grants and fellowships, and stakeholder views, by research type (for periods 1999–2006 and 2010–2017).

Stakeholders’ views regarding future priorities remained consistent across the two periods. Most commonly, stakeholders indicated that intervention studies should be given the highest priority (37%), as they had in the previous study (40%). They gave somewhat lesser emphasis to epidemiological studies (32%), which was also consistent with the views of their predecessors (36%). Figure 1 outlines key study findings regarding current and future research priorities identified across the 1999–2006 and 2010–2017 periods.

Two-sample tests of proportion (supplementary Table S2) confirmed patterns of relative stability in the proportion of research types across the two time periods, both for published journal articles and stakeholder views. Most observed -differences for these two data sources were comparatively small and did not reach statistical significance, with the only exceptions being small but significant increases of 6.1% (*p* = 0.001) in published assessment studies and of 5.5% (*p* = 0.002) in other studies for stakeholder views. By contrast, we observed large effects in terms of changes in the proportion of research types funded through grants and fellowships, with a 12.5% (*p* = 0.266) increase in epidemiological studies and a 21.9% (*p* = 0.075) decrease in intervention studies. However, when we tested these changes, none reached statistical significance. This is most likely due to the much smaller sample size involved in funded grants and fellowships (n = 36 for each time period), compared to other data sources.

## 4. Discussion

Although rising figures of peer-reviewed publications and grants and fellowship funding reflect positively on the growing recognition of suicide as a major public health issue in Australia, they arguably still fall short of being commensurate with the high individual, societal, and economic burden of suicide.

This study provides some guidance as to the direction of future Australian suicide prevention research. Our key findings indicate that despite the fact that stakeholders were calling for a greater emphasis on intervention studies in 2006, current publications and grants and fellowships suggest that priority is still being given to epidemiological studies. If anything, the situation has deteriorated; substantially less grant and fellowship funding appears to have been channelled towards intervention studies in recent times. Stakeholders of today strongly reinforce the view that future priority should be given to funding intervention studies. Our collective understanding of what works (and what does not work) in suicide prevention is still insufficient, and it is crucial that we bolster this understanding if we are to move the suicide prevention field forward [5]. It is only through examining the effectiveness—and ideally the cost-effectiveness—of interventions that our knowledge in this area will increase.

Reasons for the underrepresentation of intervention studies in research funding schemes and literature primarily include practical methodological and ethical challenges, which can hinder vital intervention research from being conducted in the first place [2]. Methodological challenges in conducting ‘gold standard’ randomised controlled trials relate to longer study timeframes, the need for sufficiently large sample sizes, adequate control groups, proper randomisation, and access to relevant outcomes data. In examining the effectiveness of universal interventions, for example, it may therefore not always be possible to select appropriate control groups or to adopt randomised controlled designs. Ethical challenges in conducting suicide prevention research include human research ethics committee concerns regarding the involvement of those at heightened risk of suicide in research, and reservations about withholding potentially effective or life-saving treatments from participants in control groups. Publication bias can further impede the publication of intervention studies which produce null (rather than positive) findings.

Strategies to overcome existing research barriers which are amenable to change therefore warrant greater attention in future. Such strategies include, amongst others, the use of multi-site studies to boost sample sizes, and the adoption of the next-most-rigorous study design in instances where the gold standard may not be feasible. Further educative work will be required with human research ethics committees, funding bodies, and the wider suicide prevention community in relation to the existing dearth and merit of intervention research, and in terms of sharing and increasing the availability of best-practice research ethics protocols among those conducting suicide prevention research.

Our examination of current priorities was limited by our reliance on publicly available information from key competitive research funding bodies and a focus on the peer-reviewed (rather than grey) literature. Future studies could widen the scope by including standard grey literature databases and other funding sources. Our review of future priorities was limited by a recruitment strategy that may have had inherent biases. We generally sought to recruit those target groups that we considered to have an inherent interest in suicide prevention research and that were included in our earlier study, except for some cases in which we targeted current equivalents of original groups that no longer existed. Notwithstanding the comprehensive recruitment strategy, varying resulting response rates, unknown profiles of non-responders, and varying degrees of knowledge about suicide prevention research among our target groups suggest that some caution should be exercised in generalising the views of questionnaire respondents to other stakeholders. While the small sample size for funded grants and fellowships precluded observed changes from reaching statistical significance, we are confident that these reflect real shifts in funding priorities.

Key study findings resonate with previous international research indicating a paucity of intervention studies globally [6] and highlight the need to establish research priorities for suicide prevention internationally.

## 5. Conclusions

To effectively address the major public health concern of suicide, it is crucial that we strengthen the evidence base for what does and does not work in suicide prevention. We have strong evidence from numerous epidemiological studies about the rates of and risk factors for suicide, and it is now time to turn our attention to testing interventions to lower the overall rates and mitigate the risk for particular groups. Mechanisms to support future intervention research in the field of suicide prevention are likely to lead to significant gains in knowledge and population health.

## Supplementary Materials

The following are available online at http://www.mdpi.com/1660-4601/15/4/807/s1, Table S1: Key Stakeholder Target Groups and Recruitment Strategy, Table S2: Percentage difference in the proportion of research types across two time periods (1999–2006 and 2010–2017).
