# Supplementary material for: Research Priorities in Suicide Prevention: Review of Australian Research from 2010–2017 Highlights Continued Need for Intervention Research
Source: Int J Environ Res Public Health. 2018 Apr 20;15(4):807. doi: 10.3390/ijerph15040807 (PMC5923849; doi:10.3390/ijerph15040807)
Supplement: Supplementary file 1 [file ijerph-15-00807-s001.zip › Table S1.docx]

**Supplementary Table S1. Key Stakeholder Target Groups and Recruitment Strategy**

| **PRIMARY ROLE IN RELATION TO SUICIDE PREVENTION RESEARCH ^a^** | **TARGET GROUP** | **RECRUITMENT STRATEGY** | **RECRUITMENT TARGET & RESPONSE (RATE) BY PRIMARY GROUP AFFILIATION ^b^** |
| --- | --- | --- | --- |
| People who conduct suicide prevention research | Researchers | Identified by study team from list of Australian first and/or corresponding authors of peer-reviewed suicide prevention journal articles published between 2010 and 2017, for whom current email addresses were available. Approach made directly by study team, via email with web-link to questionnaire. Invited to complete web-based questionnaire. Two reminders sent. | Target N=191  Response (rate): 62 (32%) |
| People who use suicide prevention research (in clinical practice) | Psychiatrists | Study invitation (including web-link to questionnaire) circulated to the Royal Australian and New Zealand College of Psychiatrists (RANZCP) membership base via Psyche newsletter. No reminder sent. | Target N (n/a)  Response (rate): 3 (n/a) |
|  | General practitioners | Study invitation (including web-link to questionnaire) circulated to members of the General Practice Mental Health Standards Collaboration (GPMHSC) via E-newsletter (goes out to GPs with an interest in mental health). No reminder sent. | Target N (n/a)  Response (rate): 16 (n/a) |
|  | Psychologists | Study invitation (including web-link to questionnaire) circulated via Australian Psychological Society (APS) newsletter, and posted on APS Research opportunities webpage (open to APS members and wider public). One reminder sent. | Target N (n/a)  Response (rate): 114 (n/a) |
|  | Mental health nurses | Study invitation (including web-link to questionnaire) circulated via (Australian College of Mental Health Nursing (ACMHN) newsletter, posted on ACMHN webpage (open to ACMHN members and wider public) and on the ACMHN Facebook page. No reminder sent. | Target N (n/a)  Response (rate): 36 (n/a) |
|  | Alumni of the Australian Institute for Suicide Research and Prevention (AISRAP) | Identified and invited by AISRAP, via email with web-link to questionnaire. One reminder sent. | Target N=260  Response (rate): 21 (8%) |
| People who use suicide prevention research (in policy-making/planning activities) | Members of the Australian Advisory Group on Suicide Prevention (AAGSP) | Identified and invited by National Mental Health Commission (NMHC) staff, via email with web-link to questionnaire. No reminder sent. | Target N=22  Response (rate): 1 (5%) |
|  | Members of the Mental Health, Drug and Alcohol Principal Committee (MHDAPC) | Identified by MHDAPC Secretariat, who emailed study invitation (including web-link to questionnaire) to MHDAPC members. One reminder sent. | Target N=9  Response (rate): 8 (89%) |
|  | Commonwealth and state/territory senior bureaucrats with responsibility for suicide prevention | Identified by MHDAPC Secretariat. Approach made directly by study team, via email with web-link to questionnaire. One reminder sent. | Target N=7  Response (rate): 3 (43%) |
|  | Primary Health Network (PHN) Suicide Prevention Managers | Identified and invited directly by study team, via email with web-link to questionnaire. Two reminders sent. | Target N=33  Response (rate): 10 (30%) |
| People involved in funding suicide prevention research | Members of the Australian Rotary Health Research Committee (ARH RC) | Identified by ARH RC. Approach made directly by study team, via email with web-link to questionnaire. Two reminders sent. | Target N=10  Response (rate): 3 (30%) |
|  | Members of the Society for Mental Health Research Executive Committee (SMHR EC) | Identified and invited directly by study team, via email with web-link to questionnaire. Two reminders sent. | Target N=14  Response (rate): 4 (29%) |
| People who are affected by suicide and/or provide advocacy for those who have been affected by suicide | Members of Suicide Prevention Australia’s Speakers Bureau (SPA SB) | Identified and invited by SPA staff, via email with web-link to questionnaire. Two reminders sent. | Target N=75  Response (rate): 10 (13%) |
|  | Members of Suicide Prevention Australia (SPA) | Identified and invited by SPA staff, via email with web-link to questionnaire. Two reminders sent. | Target N=161  Response (rate): 62 (39%) |
|  | Members of Roses in the Ocean (RITO) | Identified by RITO. Approach made directly by study team, via email with web-link to questionnaire. Two reminders sent. | Target N=100  Response (rate): 37 (37%) |

a. Showing the primary role of stakeholder groups in relation to suicide prevention research. It is acknowledged that some target groups may play additional roles, and that individual group members may be affiliated with more than one target group.

b. Showing response (rates) by primary group affiliation for which a denominator was available. These groups were typically those invited via email (either by us or by the organisation to which they belonged), and where the number of individuals on the email list constituted the denominator. Denominators and response rates could not be determined for groups whose members were approached in more diffuse ways (e.g., via newsletters or websites). Given that respondents may have been affiliated with more than one target group (but only ever completed the survey once in terms of their primary group affiliation), resulting response rates are likely to represent underestimates.
