# Supplementary material for: Research Priorities in Suicide Prevention: Review of Australian Research from 2010–2017 Highlights Continued Need for Intervention Research
Source: Int J Environ Res Public Health. 2018 Apr 20;15(4):807. doi: 10.3390/ijerph15040807 (PMC5923849; doi:10.3390/ijerph15040807)
Supplement: Supplementary file 1 [file ijerph-15-00807-s001.zip › Table S2.docx]

**Supplementary Table S2. Percentage difference in the proportion of research types across two time periods (1999-2006 and 2010-2017)**

|  | **Assessment**  **studies** | | **Epidemiology**  **studies** | | **Intervention**  **studies** | | **Evaluation**  **studies** | | **Biological**  **studies** | | **Social science**  **studies** | | **Other**  **studies** | | |
| --- | --- | --- | --- | --- | --- | --- | --- | --- | --- | --- | --- | --- | --- | --- | --- |
|  | ∆ (in %)  (95% CI) | *p* | ∆ (in %)  (95% CI) | *p* | ∆ (in %)  (95% CI) | *p* | ∆ in %  (95% CI) | *p* | ∆ (in %)  (95% CI) | *p* | ∆ (in %)  (95% CI) | *p* | ∆ (in %)  (95% CI) | *p* |  |
| **Journal**  **Articles** | 6.05  (3.20, 8.90) | .001 | -3.29  (-4.54, 11.12) | .409 | -3.53  (-9.43, 2.37) | .230 | -3.39  (-7.52, 0.74) | .087 | -0.41  (-2.71, 1.89) | .720 | -0.94  (-4.26, 2.38) | .569 | -1.06  (-5.36, 3.24) | .623 |  |
| **Stakeholder**  **Views** | 1.42  (-2.52, 5.36) | .493 | -3.99  (-11.85, 3.87) | .316 | -2.93  (-11.02, 5.16) | .476 | 1.89  (-2.61, 6.39) | .424 | 0.53  (-2.05, 3.11) | .695 | -2.42  (-6.63, 1.79) | .237 | 5.49  (2.75, 8.23) | .002 |  |
| **Grants and Fellowships** | 3.12  (-7.21, 13.45) | .555 | 12.50  (-9.32, 34.32) | .266 | -21.87  (-45.33, 1.59) | .075 | 10.94  (-1.30, 23.18) | .087 | -6.25  (-14.64, 2.14) | .151 | 4.69  (-12.69, 22.07) | .598 | -3.13  (-9.16, 2.90) | .313 |  |

Note. Based on two-sample tests of proportion.
